# Association of Spousal Diabetes Status and Ideal Cardiovascular Health Metrics With Risk of Incident Diabetes Among Chinese Adults

**DOI:** 10.1001/jamanetworkopen.2023.19038

**Published:** 2023-06-23

**Authors:** Zhiyun Zhao, Qiuyu Cao, Jieli Lu, Hong Lin, Zhengnan Gao, Min Xu, Yu Xu, Tiange Wang, Mian Li, Yuhong Chen, Shuangyuan Wang, Tianshu Zeng, Ruying Hu, Xuefeng Yu, Gang Chen, Qing Su, Yiming Mu, Lulu Chen, Xulei Tang, Li Yan, Guijun Qin, Qin Wan, Guixia Wang, Feixia Shen, Zuojie Luo, Yingfen Qin, Li Chen, Yanan Huo, Qiang Li, Zhen Ye, Yinfei Zhang, Chao Liu, Youmin Wang, Shengli Wu, Tao Yang, Huacong Deng, Jiajun Zhao, Lixin Shi, Guang Ning, Weiqing Wang, Yufang Bi

**Affiliations:** 1 Department of Endocrine and Metabolic Diseases, Shanghai Institute of Endocrine and Metabolic Diseases, Ruijin Hospital, Shanghai Jiao Tong University School of Medicine, Shanghai, China; 2 Shanghai National Clinical Research Center for Metabolic Diseases, Key Laboratory for Endocrine and Metabolic Diseases of the National Health Commission of the PR China, Shanghai Key Laboratory for Endocrine Tumor, Ruijin Hospital, Shanghai Jiao Tong University School of Medicine, Shanghai, China; 3 Dalian Municipal Central Hospital, Dalian, China; 4 Union Hospital, Tongji Medical College, Huazhong University of Science and Technology, Wuhan, China; 5 Zhejiang Provincial Center for Disease Control and Prevention, Hangzhou, China; 6 Tongji Hospital, Tongji Medical College, Huazhong University of Science and Technology, Wuhan, China; 7 Fujian Provincial Hospital, Fujian Medical University, Fuzhou, China; 8 Xinhua Hospital Affiliated to Shanghai Jiao Tong University School of Medicine, Shanghai, China; 9 Chinese People’s Liberation Army General Hospital, Beijing, China; 10 The First Hospital of Lanzhou University, Lanzhou, China; 11 Sun Yat-sen Memorial Hospital, Sun Yat-sen University, Guangzhou, China; 12 The First Affiliated Hospital of Zhengzhou University, Zhengzhou, China; 13 The Affiliated Hospital of Southwest Medical University, Luzhou, China; 14 The First Hospital of Jilin University, Changchun, China; 15 The First Affiliated Hospital of Wenzhou Medical University, Wenzhou, China; 16 The First Affiliated Hospital of Guangxi Medical University, Nanning, China; 17 Qilu Hospital of Shandong University, Jinan, China; 18 Jiangxi Provincial People’s Hospital Affiliated to Nanchang University, Nanchang, China; 19 The Second Affiliated Hospital of Harbin Medical University, Harbin, China; 20 Central Hospital of Shanghai Jiading District, Shanghai, China; 21 Jiangsu Province Hospital on Integration of Chinese and Western Medicine, Nanjing, China; 22 The First Affiliated Hospital of Anhui Medical University, Hefei, China; 23 Karamay Municipal People’s Hospital, Xinjiang, China; 24 The First Affiliated Hospital of Nanjing Medical University, Nanjing, China; 25 The First Affiliated Hospital of Chongqing Medical University, Chongqing, China; 26 Shandong Provincial Hospital Affiliated to Shandong University, Jinan, China; 27 Guiqian International General Hospital, Guiyang, China

## Abstract

**Importance:**

Spouses share common socioeconomic, environmental, and lifestyle factors, and multiple studies have found that spousal diabetes status was associated with diabetes prevalence. But the association of spousal diabetes status and ideal cardiovascular health metrics (ICVHMs) assessed by the American Heart Association’s Life’s Essential 8 measures with incident diabetes has not been comprehensively characterized, especially in large-scale cohort studies.

**Objective:**

To explore the association of spousal diabetes status and cardiovascular health metrics with risk of incident diabetes in Chinese adults.

**Design, Setting, and Participants:**

This cohort study included individuals in the China Cardiovascular Disease and Cancer Cohort without diabetes who underwent baseline and follow-up glucose measurements and had spouses with baseline glucose measurements. The data were collected in January 2011 to December 2012 and March 2014 to December 2016. The spousal study had a mean (SD) follow-up of 3.6 (0.9) years (median [IQR], 3.2 [2.9-4.5] years). Statistical analysis was performed from July to November 2022.

**Exposure:**

Spousal diabetes status was diagnosed according to the 2010 American Diabetes Association (ADA) criteria. All participants provided detailed clinical, sociodemographic, and lifestyle information included in cardiovascular health metrics.

**Main Outcomes and Measures:**

Incident diabetes, diagnosed according to 2010 ADA criteria.

**Results:**

Overall, 34 821 individuals were included, with a mean (SD) age of 56.4 (8.3) years and 16 699 (48.0%) male participants. Spousal diabetes diagnosis was associated with an increased risk of incident diabetes (hazard ratio [HR], 1.15; 95% CI, 1.03-1.30). Furthermore, participants whose spouses had uncontrolled glycated hemoglobin (HbA_1c_) had a higher risk of diabetes (HR, 1.20; 95% CI, 1.04-1.39) but the risk of diabetes in participants whose spouses had controlled HbA_1c_ did not increase significantly (HR, 1.10; 95% CI, 0.92-1.30). Moreover, this association varied with composite cardiovascular health status. Diabetes risk in individuals who had poor cardiovascular health status (<4 ICVHMs) was associated with spousal diabetes status (3 ICVHMs: HR, 1.50; 95% CI, 1.15-1.97), while diabetes risk in individuals who had intermediate to ideal cardiovascular health status (≥4 ICVHMs) was not associated with it (4 ICVHMs: HR, 1.01; 95% CI, 0.69-1.50).

**Conclusions and Relevance:**

In this study, spousal diabetes diagnosis with uncontrolled HbA_1c_ level was associated with increased risk of incident diabetes, but strict management of spousal HbA_1c_ level and improving ICVHM profiles may attenuate the association of spousal diabetes status with diabetes risk. These findings suggest the potential benefit of couple-based lifestyle or pharmaceutical interventions for diabetes.

## Key Points

**Question:**

Is there an association between spousal diabetes status, ideal cardiovascular health metrics, and incident diabetes?

**Findings:**

In this cohort study with 34 821 Chinese adults, spousal diabetes status with uncontrolled glycated hemoglobin level was independently associated with increased risk of incident diabetes. Achievement of ideal cardiovascular health metrics attenuated this risk.

**Meaning:**

These findings suggest the potential benefit of couple-based lifestyle or pharmaceutical interventions for diabetes.

## Teaser

This cohort study explores the association of spousal diabetes status and cardiovascular health metrics with risk of incident diabetes in Chinese adults.

## Introduction

The diabetes epidemic represents an escalating challenge worldwide.^1^ China also has witnessed a substantial increasing trend of diabetes over the past decades.^2^ The prevalence of diabetes in China has exceeded 11% since 2010, representing more than 100 million adults with diabetes, which indicated the urgent need for diabetes prevention and control not only globally, but especially in China.^3^

A family history of diabetes is a critical contributor to diabetes risk and is frequently involved in diabetes risk scores.^4,5^ Previous epidemiologic studies have observed evident aggregation of patients with diabetes within households.^6,7^ This phenomenon could be attributable to heritable factors, but much of it remains yet to be explained, which may be captured by studies on spousal concordance. Spouses are generally genetically unrelated, yet they may share common nonheritable factors, such as living environments, social habits, and health behaviors.^8^ For this reason, cohabiting couples are often at risk of the same diseases, including cardiovascular disease, metabolic syndrome and diabetes.^9^

Previous studies suggested that individuals with a spouse with diabetes were at an increased risk of developing type 2 diabetes,^10,11^ whereas some studies did not find a significant spousal correlation of diabetes prevalence.^12^ The heterogeneity of research conclusions may be due to the difference in study design, participants, follow-up periods, and methods for diabetes diagnosis. Most of those studies were cross-sectional,^12^ had a small sample size,^11^ or indirectly ascertained diabetes (based on participants’ self-reports or electronic medical records rather than systematic glucose testing).^13^ Thus, evidence from large-scale cohort studies using standard criteria for diabetes diagnosis is still required.

Moreover, it remains unclear how to avoid or reduce the association of spousal diabetes status with individuals’ risk of diabetes. In 2010, the American Heart Association (AHA) proposed 7 ideal cardiovascular health metrics (ICVHMs) to guide healthy behaviors and ideal metabolic parameters to lower cardiovascular disease (CVD) risk.^14^ Recently, the AHA updated the definition of ICVHMs to Life’s Essential 8, which included diet, physical activity, nicotine exposure, sleep health, body mass index (BMI [calculated as weight in kilograms divided by height in meters squared]), blood pressure, blood glucose levels, and blood lipid levels.^15^ Multiple previous studies indicated that the components of ICVHMs were closely associated with a reduction of diabetes incidence,^16,17^ but the association of comprehensive ICVHM status with the interaction between spouses’ metabolic status requires further investigation.

In the cross-sectional analysis we launched in 2016, spouses of individuals with diabetes were found to have a higher prevalence of diabetes, obesity, metabolic syndrome, and CVD.^18^ We aim to further investigate the diabetes incidence of individuals whose spouses were diagnosed with diabetes and the association of the comprehensive ICVHM profile and spousal diabetes status with diabetes risk to examine the potential value of couple-based diabetes interventions.

## Methods

### Study Design and Population

The China Cardiometabolic Disease and Cancer Cohort (4C) Study is a multicenter, nationwide, prospective cohort study consisting of community-dwelling adults aged 40 years or older. The study protocol and informed consent were approved by the Committee on Human Research at Ruijin Hospital, affiliated with Shanghai Jiao Tong University School of Medicine, Shanghai, China. All participants provided written informed consent. This study followed the Strengthening the Reporting of Observational Studies in Epidemiology (STROBE) reporting guideline.

The details of the 4C Study design were described previously,^19,20^ and more details of study visits and statistical methods are included in the eAppendix in Supplement 1. Among 193 846 participants examined at baseline in 2011 to 2012, 32 196 couples (64 392 individuals) participated, and 58 254 individuals (90.5%) attended the in-person follow-up visit in 2014 to 2016, with a mean (SD) follow-up time of 3.6 (0.9) years. The median (IQR) follow-up time was 3.2 (2.9-4.5) years. Overall, 9.5% of the baseline participants (6138) were lost to follow-up. Of the remaining 58 254 individuals, 40 010 individuals without diabetes were selected by excluding 13 166 participants with diagnosed or screen-detected diabetes at baseline and 5078 participants with indeterminate diabetes status due to missing baseline information on plasma glucose measurement of their own or their spouse’s. After excluding 5189 participants who did not complete fasting blood glucose or oral glucose tolerance test (OGTT) measurements at follow-up, 34 821 individuals entered the current analysis (eFigure 1 in Supplement 1). The distribution of study participants is shown in eFigure 2 in Supplement 1.

### Data Collection

Data collection was conducted in local community clinics by trained staff according to standard protocols at baseline and follow-up visits. A questionnaire collecting information about demographic characteristics, lifestyle patterns (including smoking, drinking habits, physical activity, dietary habits, and sleep patterns), chronic diseases history, and medication usage was administered by trained personnel. Skilled nurses measured height, weight, and blood pressure of participants according to standard protocols. All participants underwent an OGTT after an overnight fast for at least 10 hours, and blood samples were collected at 0 and 2 hours during the test. Plasma fasting glucose (FPG) and 2-hour postload glucose (2h-PG) concentrations were evaluated at local hospitals using the glucose oxidase or hexokinase method. The levels of glycated hemoglobin (HbA_1c_) and lipids were examined at the central laboratory. HbA_1c_ was tested using finger capillary whole blood by high-performance liquid chromatography (VARIANT II Systems [Bio-Rad]). Serum total cholesterol, low-density lipoprotein cholesterol (LDL-C), high-density lipoprotein cholesterol (HDL-C). and triglycerides were tested using an autoanalyzer (Abbott Laboratories) at the central laboratory. Data types and definitions of covariates involved in the study are listed in eTable 1 in Supplement 1.

### Definition of ICVHMs

The updated Life’s Essential 8 proposed by the AHA included a point scoring system for each metric (ranging from 0-100 points).^15^ Accordingly, 7 metrics reaching 100 points were defined as ICVHMs: ideal smoking status, physical activity at goal, healthy sleep habits, ideal BMI (<23), untreated blood pressure of less than 120 mm Hg systolic blood pressure and less than 80 mm Hg diastolic blood pressure, untreated non–HDL-C of less than 130 mg/dL (to convert to millimoles per liter, multiply by 0.0259), and untreated fasting plasma glucose of less than 100 mg/dL (to convert to millimoles per liter, multiply by 0.0555) or HbA_1c_ of less than 5.7% (to convert to proportion of total hemoglobin, multiply by 0.01). The food frequency questionnaire of the previous 12 months was used to characterize dietary metrics and defined healthy dietary habits according to the modified components of the Mediterranean Eating Pattern for Americans (MEPA) score, which were more consistent with the dietary habits of the Chinese population (eTable 2 in Supplement 1).

### Diabetes Ascertainment

According to the 2010 American Diabetes Association criteria,^21^ incident diabetes was diagnosed if (1) fasting plasma glucose was 126.1 mg/dL or greater, and/or (2) 2h-PG was 200.0 mg/dL or greater, and/or (3) HbA_1c_ level was 6.5% (≥48 mmol/mol) or greater. Self-reported diagnosis by clinicians of diabetes were also included.

### Statistical Analysis

The baseline characteristics of individuals according to their spousal diabetes status were presented as proportions in categorical variables, means and SDs in normally distributed variables, and medians and IQRs in nonnormally distributed variables. One-way analysis of variance was used to compare continuous variables, and χ^2^ tests were used to compare categorical variables.

The cumulative incidence of diabetes was calculated for a mean follow-up of 3.6 years. The association of incident diabetes with spouse’s diabetes status was examined using Cox proportional hazard analysis. Participants who did not have spouses with diabetes at baseline were the reference group. To investigate whether spousal HbA_1c_ level affected the association, the analysis was further performed in subgroups categorized by HbA_1c_ level of less than 7.0% and 7.0% or greater. Model 1 was adjusted for age, sex, high school education or greater, family history of diabetes, local personal income, and urban or not. Model 2 was further adjusted for obesity, hypertension, dyslipidemia, and prediabetes status based on model 1. Model 3 was further adjusted for diet score, physical activity, sleep, and smoking status based on model 2. Model 4 was further adjusted for 7 spousal ICVHM factors (obesity [BMI ≥23] hypertension, dyslipidemia, diet, physical activity, sleep and smoking status, excluding glucose status) based on model 3.

To comprehensively analyze the association of CVH status or ICVHM profile and spousal diabetes status with incident diabetes, the study population was stratified in 3 ways, according to the participant’s ICVHM components and number, the spouse’s ICVHM number, and a comparison of the numbers of ICVHMs of a participant and the spouse. The association between spousal diabetes status and incident diabetes was estimated in these strata. To compare the CVH status of the couples, 3 groups were created: (1) participant has more ICVHMs than spouse, (2) participant and spouse have same number of ICVHMs, and (3) participant has fewer ICVHMs than spouse. These 3 levels reflected the CVH status in a participant who had better, equal, or worse cardiovascular health markers than their spouse, respectively. To demonstrate possible interactions of ICVHM and spousal diabetes status in the development of diabetes, interaction terms were created using the cross products of spousal diabetes status with components or number of ICVHMs. The interaction was tested using the likelihood ratio test by comparing the full model including the interaction term with the reduced model excluding the interaction term.

Six sensitivity analyses were performed. To test sex disparities, the main analysis was performed separately in women and men (eTables 3 and 4 and eFigures 3 and 4 in Supplement 1). To examine the robustness of the results, the main analysis was performed in a model further adjusting for alcohol consumption (eTable 5 in Supplement 1) or replacing prediabetes status with HbA_1c_ level (eTable 6 in Supplement 1). The validity of the main results was also tested in multiple imputation data sets imputed for missing baseline information (eTables 7-10 in Supplement 1) or outcome (eTable 11 in Supplement 1). To address the center effect, the major analysis was performed by random-effects models (eTable 12 in Supplement 1).

SAS software version 9.4 (SAS Institute) was used for all statistical analyses from July 2022 to November 2022. All reported *P* values are nominal. Statistical significance was a 2-tailed *P* < .05.

## Results

### Baseline Characteristics of the Study Population

Overall, 34 821 individuals were included, with a mean (SD) age of 56.4 (8.3) years and 16 699 (48.0%) male participants. As shown in the Table, the group of participants whose spouses had been diagnosed with diabetes were older, had more education, and tended to live in urban areas than individuals whose spouses had not been diagnosed with diabetes. The lifestyle patterns of participants with spouses diagnosed with diabetes were generally less healthy than those whose spouses were not diagnosed with diabetes. BMI, systolic blood pressure, 2h-PG, HbA_1c_ level, and total cholesterol and LDL-C levels were higher in participants with spouses diagnosed with diabetes than in those whose spouses were not diagnosed with diabetes. There was no significant difference in FPG levels among them, possibly because elevated 2h-PG levels might develop earlier than elevated FPG levels at the early stage of metabolic disease in the Chinese population.^19^ Among participants with spouses who had uncontrolled blood glucose, we observed higher systolic blood pressure and lower HDL-C levels. The characteristics of individuals who developed and did not develop diabetes were also significantly different (eTable 13 in Supplement 1). Spouses had high concordance among most components of ICVHMs (eTable 14 in Supplement 1).

**Table.** Baseline Characteristics of Participants Without Diabetes by Spouse’s Diabetes Status

Characteristics	Participants, No. (%)			
	Spouse without previously diagnosed diabetes (n = 31 616)	Spouse with previously diagnosed diabetes^a^		
		Any glucose level (n = 3205)	HbA_1c_ <7.0% (n = 1454)	HbA_1c_ ≥7.0% (n = 1751)
Age, mean (SD), y	56.1 (8.25)	59.4 (8.20)^b^	59.8 (8.26)^b^	59.1 (8.14)^b^
Sex				
Male	15 411 (48.7)	1288 (40.2)^b^	602 (41.4)^b^	686 (39.2)^b^
Female	16 205 (51.3)	1917 (59.8)^b^	852 (58.6)^b^	1065 (60.8)^b^
Family history of diabetes	3319 (10.5)	334 (10.4)	151 (10.4)	183 (10.5)
High school education or more	11 749 (37.2)	1286 (40.1)^b^	585 (40.2)^b^	701 (40.0)^b^
Urban residence	19 429 (61.5)	2399 (74.9)^b^	1058 (72.8)^b^	1341 (76.6)^b^
Current smoker	6709 (21.2)	488 (15.2)^b^	220 (15.1)^b^	268 (15.3)^b^
Current drinker	4537 (14.4)	353 (11.0)^b^	170 (11.7)^b^	183 (10.5)^b^
Sufficient physical activity	4364 (13.8)	468 (14.6)	213 (14.7)	255 (14.6)
Diet scores, mean (SD)^c^	5.68 (2.10)	5.93 (2.14)^b^	5.88 (2.13)^b^	5.98 (2.14)^b^
Sleep duration, mean (SD), h/d	8.28 (1.50)	8.14 (1.42)^b^	8.17 (1.34)^b^	8.12 (1.48)^b^
BMI, mean (SD)	24.5 (3.46)	24.9 (3.53)^b^	24.7 (3.58)^b^	25.1 (3.48)^b^
Systolic BP, mean (SD), mm Hg	131.1 (19.4)	132.6 (19.9)	132.0 (19.5)	133.1 (20.2)^a^
Diastolic BP, mean (SD), mm Hg	78.5 (10.9)	78.1 (10.6)^a^	77.6 (10.5)^a^	78.6 (10.8)
FPG, mean (SD), mg/dL	98.02 (9.73)	98.20 (9.55)	97.84 (9.91)	98.38 (9.37)
2h-PG, mean (SD), mg/dL	121.98 (30.45)	123.96 (30.45)^b^	123.42 (30.09)^b^	124.32 (30.63)^b^
HbA_1c_, mean (SD), %	5.67 (0.38)	5.74 (0.36)^a^	5.71 (0.35)^a^	5.76 (0.37)^a^
Total cholesterol, mean (SD), mg/dL	188.42 (42.47)	191.89 (41.7)^b^	192.28 (42.08)^b^	191.12 (41.31)^b^
LDL-C, mean (SD), mg/dL	109.65 (33.2)	112.36 (32.43)^b^	112.74 (32.43)^b^	111.97 (32.43)^b^
HDL-C, mean (SD), mg/dL	50.97 (13.9)	50.58 (13.13)	50.97 (13.13)	50.19 (12.74)^b^
Triglycerides, median (IQR), mg/dL	112.39 (81.42)	116.81 (81.42)^b^	115.04 (75.22)^b^	118.58 (86.73)^b^
Obesity^d^	20 995 (66.4)	2261 (70.6)^b^	997 (68.6)	1264 (72.2)^b^
Hypertension	12 847 (40.6)	1416 (44.2)^b^	633 (43.5)^b^	783 (44.7)^b^
Dyslipidemia^e^	12 886 (40.8)	1373 (42.8)^b^	605 (41.6)	768 (43.9)^b^
Spouse’s FPG, median (IQR), mg/dL	99.1 (15.14)	140.00 (50.45)^b^	120.90 (27.93)^b^	160.54 (56.76)^b^
Spouse’s 2h-PG, median (IQR), mg/dL	124.32 (50.45)	239.64 (112.61)^b^	196.4 (83.78)^b^	279.28 (112.07)^b^
Spouse’s HbA_1c_, mean (SD), %	5.82 (0.71)	7.50 (1.61)^a^	6.28 (0.47)^b^	8.51 (1.51)^b^

Abbreviations: BP, blood pressure; BMI, body mass index (calculated as weight in kilograms divided by height in meters squared); HDL-C, high-density lipoprotein cholesterol; LDL-C, low-density lipoprotein cholesterol; FPG, fasting blood glucose; 2h-PG, 2-hour postload glucose; HbA_1c_, glycated hemoglobin.

SI conversion factors: To convert cholesterol, HDL-C, and LDL-C to millimoles per liter, multiply by 0.0259; FPG and 2h-PG to millimoles per liter, multiply by 0.0555; and HbA_1c_ to proportion of total hemoglobin, multiply by 0.01; triglycerides to millimoles per liter, multiply by 0.0113.

^a^ Diabetes was defined by FPG of 126.1 mg/dL or greater, and/or 2h-PG of 200.0 mg/dL or greater, and/or HbA_1c_ of 6.5% or greater, and/or self-report.

^b^ *P* < .05 vs spouse without previously diagnosed diabetes.

^c^ Diet score ranged from 0 to 11, with a higher score indicating healthier diet habits.

^d^ Obesity was defined as a BMI of 23 or greater.

^e^ Dyslipidemia was defined as a total cholesterol level of 240 mg/dL or greater and/or an LDL-C level of 160 mg/dL or greater and/or HDL-C level of 40 mg/dL or greater and/or triglyceride level of 200 mg/dL or greater and/or use of lipid-lowering medications.

### Association Between Spousal Diabetes Status and Incident Diabetes

**Figure 1.**
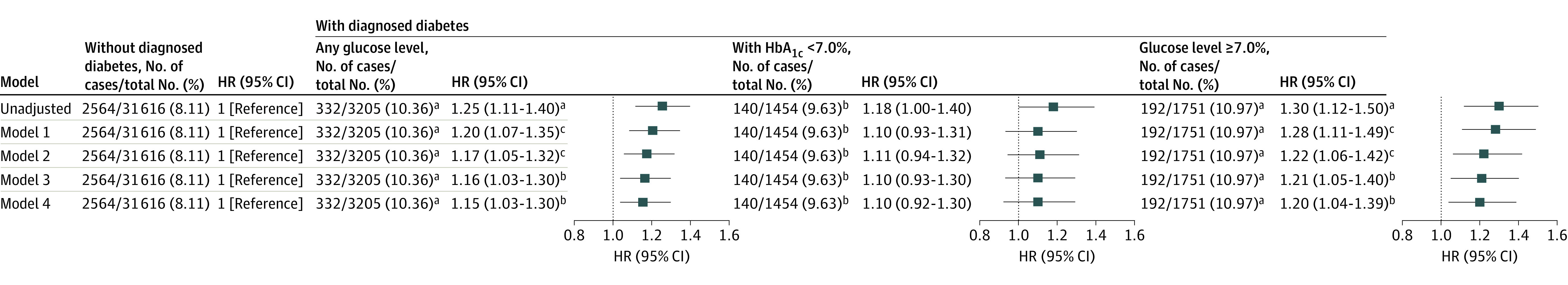
Association of Spousal Diabetes Status With Incident Diabetes Diabetes was defined by fasting plasma glucose level of 126.1 mg/dL or greater and/or a 2-hour plasma glucose level of 200.0 mg/dL or greater (to convert to millimoles per liter, multiply by 0.0555) and/or a hemoglobin A_1c_ level of 6.5% (to convert to proportion of total hemoglobin, multiply by 0.01), and/or self-report. Model 1 was adjusted for age, sex, high school education or greater, family history of diabetes, local personal income, and urban residence. Model 2 was further adjusted for obesity, hypertension, dyslipidemia and prediabetes status based on model 1. Model 3 was further adjusted for diet score, physical activity, sleep, and smoking status based on model 2. Model 4 was further adjusted for 7 spousal ICVHM factors (obesity, hypertension, dyslipidemia, diet, physical activity, sleep, and smoking status, excluding glucose status) based on model 3. Reference group was participants with spouses without previously diagnosed diabetes at baseline. HR indicates hazard ratio. ^a^*P* < .001. ^b^*P* < .05. ^c^*P* < .01.

During the follow-up of 3.6 years, 2896 individuals experienced incident diabetes (2564 [88.5%] had spouses without diabetes and 332 [11.5%] had spouses with diabetes). We analyzed the association between spousal diabetes status and incident diabetes (Figure 1). After multivariate adjustment, spousal diabetes diagnosis was associated with a 15% higher risk of incident diabetes (hazard ratio [HR], 1.15; 95% CI, 1.03-1.30). Notably, individuals whose spouses had an HbA_1c_ level of 7.0% or greater had a 20% higher risk of incident diabetes (HR, 1.20; 95% CI, 1.04-1.39) than people without spouses diagnosed with diabetes, while the risk of incident diabetes in participants whose spouses had an HbA_1c_ level of less than 7.0% was not increased significantly (HR, 1.10; 95% CI, 0.92-1.30). The associations remained similar in the sensitivity analyses (eTables 5-12 in Supplement 1). The analysis was performed separately in women and men. When husbands had diabetes with high HbA_1c_ levels, wives tended to have a higher risk of incident diabetes (HR, 1.25; 95% CI, 1.03-1.52), but when wives had diabetes, husbands tended not to have a higher risk of diabetes (HR, 1.14; 95% CI, 0.90-1.44) (eTables 3 and 4 in Supplement 1).

### Associations of Individual ICVHMs and Spousal Diabetes Status With Incident Diabetes

**Figure 2.**
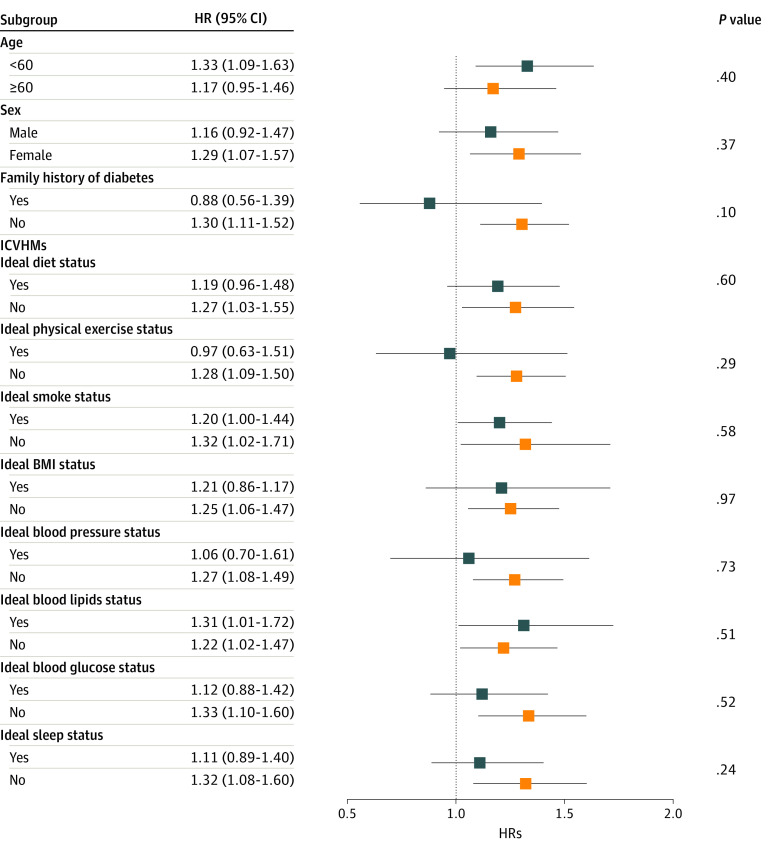
Association of Spousal Diabetes Status and Individual Ideal Cardiovascular Health Metric Components With Diabetes Incidence The model was adjusted for age, sex, high school education or above, family history of diabetes, local personal income, urban residence or not, obesity, blood pressure, blood lipids, blood glucose, diet, physical activity, sleep, and smoking status (except the current stratified factor) and 7 spousal ideal cardiovascular health metric factors (obesity, hypertension, dyslipidemia, diet, physical activity, sleep and smoking status, excluding glucose status) based on model 3 (described in the Statistical Analysis section). BMI indicates body mass index; HR, hazard ratio; ICVHM, ideal cardiovascular health metric.

**Figure 3.**
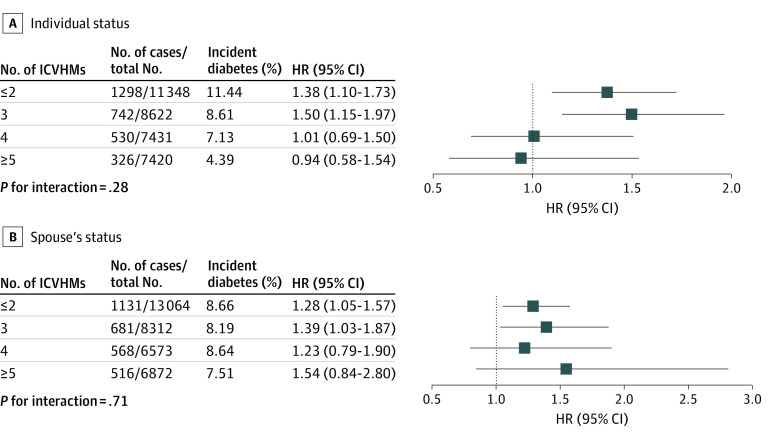
Association of Spousal Diabetes Status and Number of Individual Ideal Cardiovascular Health Metrics (ICVHMs) With Incident Diabetes The model was adjusted for age, sex, high school education or above, family history of diabetes, local personal income, and urban residence or not. HR indicates hazard ratio.

As shown in Figure 2, there was no significant interaction between individual ICVHM components and spousal diabetes status for incident diabetes, with all *P* values for interaction greater than .05. However, variation was observed in the association with different ICVHM numbers or CVH status. As shown in Figure 3A, if participants had intermediate to ideal CVH status (≥4 ICVHMs), their diabetes risk associated with spousal diabetes status would be attenuated (4 ICVHMs: HR, 1.01; 95% CI, 0.69-1.50), compared with that of individuals with poor CVH status (<4 ICVHMs) (3 ICVHMs: HR, 1.50; 95% CI, 1.15-1.97). Analysis of the composite CVH scores also showed similar results (eFigure 5 in Supplement 1).

### Associations of Spousal ICVHMs and Spousal Diabetes Status With Incident Diabetes

As Figure 3B shows, the risk of incident diabetes attributable to spousal diabetes diagnosis varied with spousal CVH status. If the spouses with diabetes had intermediate to ideal CVH status (≥4 ICVHMs), the association between spousal diabetes diagnosis and incident diabetes was attenuated (4 ICVHMs: HR, 1.23; 95% CI, 0.79-1.90), but the association persisted if spouses had poor CVH status (<4 ICVHMs) (3 ICVHMs: HR, 1.39; 95% CI, 1.03-1.87). The results were similar in spouses with different composite CVH scores (eFigure 6 in Supplement 1).

### Associations of Comparison of Numbers of ICVHMs Between Couples and Spousal Diabetes Status With Incident Diabetes

**Figure 4.**
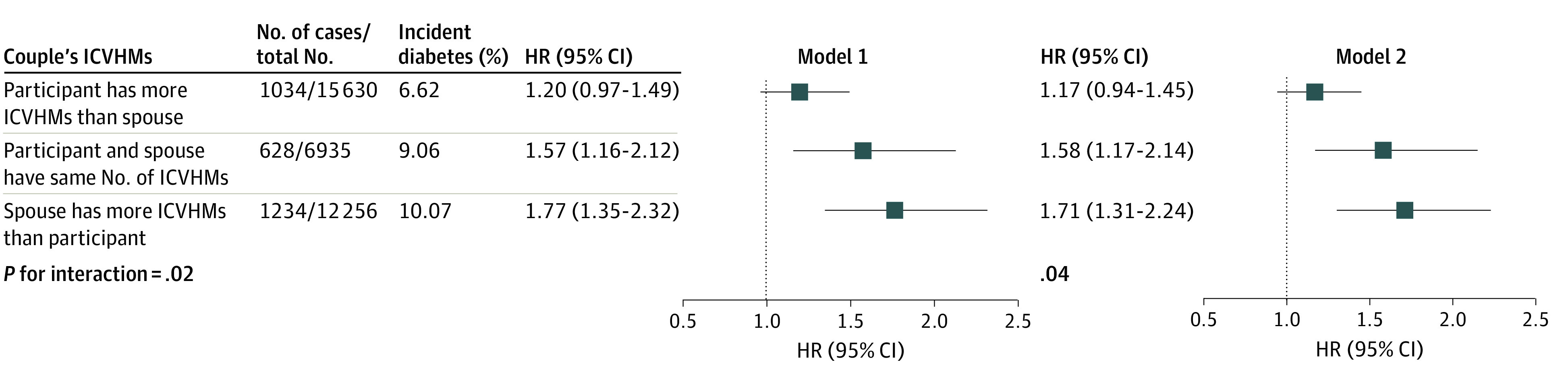
Association of Comparison of Numbers of Individual Ideal Cardiovascular Health Metrics (ICVHMs) Between Participants and Spouses and Spousal Diabetes Status With Incident Diabetes Model 1 was unadjusted; model 2 was adjusted for high school education or above, family history of diabetes, local personal income, and urban residence or not. HR indicates hazard ratio.

As Figure 4 shows, if a participant had better CVH status or a higher number of ICVHMs than their spouse, spousal diagnosis of diabetes was not significantly associated with risk of diabetes (HR, 1.17; 95% CI, 0.94-1.45); however, if a participant had an equal number of or fewer ICVHMs than their spouse, spousal diagnosis of diabetes was associated with incident diabetes (equal ICVHMs: HR, 1.58; 95% CI, 1.17-2.14; fewer ICVHMs: HR, 1.71; 95% CI, 1.31-2.24), the interaction of which was also significant (*P* = .04). The results were similar with results using categories of composite CVH scores (eFigure 7 in Supplement 1). The variation of association was more evident in wives than in husbands, with a *P* for interaction of .02 in wives compared with .24 in husbands (eFigures 3 and 4 in Supplement 1).

## Discussion

In this nationwide prospective cohort study in China, we found that incident diabetes was associated with spousal diabetes status, and the risk was higher if spouses had HbA_1c_ levels of 7.0% or greater. However, this association varied with the composite CVH status of husbands and wives, assessed by Life’s Essential 8. The association between spousal diabetes diagnosis and incident diabetes was attenuated if either the participant or spouse had intermediate to ideal CVH status, or at least 4 ICVHMs. Better CVH status in participants than in their spouses also attenuated the association. To our knowledge, our study is one of the large-scale population-based cohort studies in the East Asian population to reveal the association of spousal diabetes status and composite cardiometabolic profiles with incident diabetes, diagnosed by comprehensive measurements of FPG, 2h-PG, and HbA_1c_ levels.

The association of spousal diabetes status with incident diabetes has been studied extensively. The Atherosclerosis Risk in Communities (ARIC) study found a positive association between spousal diabetes status and the development of diabetes (HR, 1.20; 95% CI, 1.02-1.41).^10^ The authors also performed a meta-analysis of 17 studies, and the association remained significant. In a cross-sectional analysis we conducted in 2016, we also observed that having a spouse diagnosed with diabetes was associated with a 33% higher risk of diabetes in men and 35% in women.^18^ The current study confirmed in a large-scale Chinese cohort that spousal diabetes was associated with a 15% higher risk of incident diabetes after adjustment of important covariates, which is in line with previous findings. There are several possible mechanisms for the association.^10,22^ One is assortative mating. Another refers to spouses converging in their lifestyles over marriage due to engaging in similar activities and habits. Further research on underlying mechanisms is needed.

Moreover, our study found that the risk of incident diabetes increased 20% when spouses had uncontrolled HbA_1c_ levels but did not increase if spouses had adequate HbA_1c_ control. The study suggested that good control of blood glucose could reduce rates of diabetes-related comorbidity and protect spouses of patients with diabetes from increased risk of diabetes. Strict control of glucose levels in patients with diabetes appears to be essential to benefit both the patients and their spouses.

The study also found that the diabetes risk attributable to spousal diabetes diagnosis was associated with healthy behaviors and metabolic profiles in couples. To reduce CVD morbidity and mortality, the AHA defined 7 components of ideal cardiovascular health in 2010 and updated the ICVHM to 8 components recently, named Life’s Essential 8. The ARIC study defined participants with 0 to 2, 3 to 4, and 5 or more ICVHMs as having poor, intermediate, and ideal cardiovascular health, respectively.^23^ It was suggested in multiple studies that these metrics were associated with lower diabetes incidence.^16,17^ However, few longitudinal studies of couples have investigated whether the composite CVH profile was associated with spousal similarities in diabetes risk. In our study, the association between spousal diabetes diagnosis and incident diabetes varied with ICVHMs in couples. Our results support the potential benefits of couple-based lifestyle or pharmacologic interventions to promote metabolic health. It is advised that both individuals and their spouses with diabetes adopt a healthier lifestyle to reduce the diabetes risk in individuals. Recently, a randomized clinical trial (PROLIFIC) in the families of individuals with premature coronary heart disease found that a family-based CVD risk reduction intervention could effectively reduce total CVD risk in those patients.^24^ Clinical trials are required to examine the efficacy of couple-targeted collaborative lifestyle and metabolic modification programs for type 2 diabetes.^25^

Furthermore, our study found that the association of spousal diabetes diagnosis with incident diabetes in wives was stronger than in husbands. The variation in the association by the difference in numbers of ICVHMs between couples was more evident in wives than in husbands as well. One possible reason that there was no association observed in husbands could be the relatively small sample size and lack of statistical power. Another reason could be the underlying difference between wives and husbands. Our findings are consistent with previous studies performed in the United Kingdom and the Netherlands, showing that wives were more likely to be impacted by their spouses’ chronic status than husbands.^26,27^ It could be explained by the different roles of husbands and wives in Chinese families.^28^ Wives may be more dependent on their spouses regarding lifestyles and health management than husbands. Further research on sex disparities is required.

Our study had several strengths. First, the conclusion of the study is derived from a large cohort representative of the general Chinese population. Second, comprehensive assessments of socioeconomic, behavioral, and biochemical profiles in participants enabled us to investigate the association of ICVHM components with spousal diabetes.

### Limitations

There are some limitations to take into consideration. First, we were unable to determine how the length of marriage or cohabitation affected the reported association because we did not have such information. However, previous studies did not find significant associations between duration of marriage and spousal risk of diabetes.^9,29^ The interaction of the duration of marriage with the association requires further investigation. Second, only spousal diabetes and ICVHMs measured at baseline were analyzed. These factors might have changed during the follow-up, and couples might have separated. Third, the 3.6-year follow-up time was relatively short. Therefore, our models were adjusted for multiple confounders, stratified analyses were performed according to individual and spousal ICVHM profiles, and the previously described associations were observed. However, the underlying mechanisms and causal relationships need to be examined by studies with longer follow-up times. Additionally, only heterosexual legally married couples were included in the study.

## Conclusions

This nationwide prospective study found that spousal diabetes status was associated with an increased risk of incident diabetes, and the risk associated with spousal uncontrolled glucose level was even higher. However, the association between spousal diabetes diagnosis and incident diabetes was attenuated if either the participant or spouse had ideal ICVHM profiles.

## Notes

Supplement 1. **eAppendix.** Supplementary Methods **eTable 1.** Data Types and Definitions of Covariates Involved in the Study **eTable 2.** Definitions of ICVHM **eTable 3.** Association Between Husband’s Diabetes Diagnosis and Incident Diabetes in Women **eTable 4.** Association Between Wife’s Diabetes Diagnosis and Incident Diabetes in Men **eTable 5.** Association Between Spousal Diabetes Diagnosis and Incident Diabetes in Models Further Adjusted for Alcohol Consumption **eTable 6.** Association Between Spousal Diabetes Diagnosis and Incident Diabetes in Models Replacing Prediabetes Status with Baseline HbA1c Level **eTable 7.** Association Between Spousal Diabetes Diagnosis and Incident Diabetes in Multiple Imputation Data Set Imputed for Missing Baseline Information **eTable 8.** Association of Individual ICVHMs and Spousal Diabetes Diagnosis With Incident Diabetes in Multiple Imputation Data Set Imputed for Missing Baseline Information **eTable 9.** Association of Spousal ICVHMs and Spousal Diabetes Diagnosis With Incident Diabetes in Multiple Imputation Data Set Imputed for Missing Baseline Information **eTable 10.** Association of Comparison of Numbers of ICVHMs Between Couples and Spousal Diabetes Diagnosis With Incident Diabetes in Multiple Imputation Data Set Imputed for Missing Baseline Information **eTable 11.** Association Between Spousal Diabetes Diagnosis With Incident Diabetes in Multiple Imputation Data Set Imputed for Outcome **eTable 12.** Association Between Spousal Diabetes Diagnosis With Incident Diabetes Estimated by Random-Effects Models **eTable 13.** Baseline Characteristics of Individuals Categorized by Diabetes Diagnosis at Follow-up and Spousal Diabetes Status at Baseline **eTable 14.** Percentage of Spousal Concordance by the ICVHMs **eFigure 1.** Participant Flow Diagram **eFigure 2.** Study Sites and Participant Distribution **eFigure 3.** Association of Comparison of Numbers of ICVHMs Between Couples and Spousal Diabetes Diagnosis With Incident Diabetes in Men **eFigure 4.** Association of Comparison of Numbers of ICVHMs Between Couples and Spousal Diabetes Diagnosis With Incident Diabetes in Women **eFigure 5.** Association of Individual CVH Score Categories and Spousal Diabetes Diagnosis With Incident Diabetes **eFigure 6.** Association of Spousal CVH Score Categories and Spousal Diabetes Diagnosis With Incident Diabetes **eFigure 7.** Association of Comparison of CVH Scores Between Couples and Spousal Diabetes Diagnosis With Incident Diabetes

Supplement 2.
Data Sharing Statement
