# Supplementary material for: Association of Spousal Diabetes Status and Ideal Cardiovascular Health Metrics With Risk of Incident Diabetes Among Chinese Adults
Source: JAMA Netw Open. 2023 Jun 23;6(6):e2319038. doi: 10.1001/jamanetworkopen.2023.19038 (PMC10290251; doi:10.1001/jamanetworkopen.2023.19038)
Supplement: Supplement 1. — eAppendix. Supplementary Methods eTable 1. Data Types and Definitions of Covariates Involved in the Study eTable 2. Definitions of ICVHM eTable 3. Association Between Husband’s Diabetes Diagnosis and Incident Diabetes in Women eTable 4. Association Between Wife’s Diabetes Diagnosis and Incident Diabetes in Men eTable 5. Association Between Spousal Diabetes Diagnosis and Incident Diabetes in Models Further Adjusted for Alcohol Consumption eTable 6. Association Between Spousal Diabetes Diagnosis and Incident Diabetes in Models Replacing Prediabetes Status with Baseline HbA1c Level eTable 7. Association Between Spousal Diabetes Diagnosis and Incident Diabetes in Multiple Imputation Data Set Imputed for Missing Baseline Information eTable 8. Association of Individual ICVHMs and Spousal Diabetes Diagnosis With Incident Diabetes in Multiple Imputation Data Set Imputed for Missing Baseline Information eTable 9. Association of Spousal ICVHMs and Spousal Diabetes Diagnosis With Incident Diabetes in Multiple Imputation Data Set Imputed for Missing Baseline Information eTable 10. Association of Comparison of Numbers of ICVHMs Between Couples and Spousal Diabetes Diagnosis With Incident Diabetes in Multiple Imputation Data Set Imputed for Missing Baseline Information eTable 11. Association Between Spousal Diabetes Diagnosis With Incident Diabetes in Multiple Imputation Data Set Imputed for Outcome eTable 12. Association Between Spousal Diabetes Diagnosis With Incident Diabetes Estimated by Random-Effects Models eTable 13. Baseline Characteristics of Individuals Categorized by Diabetes Diagnosis at Follow-up and Spousal Diabetes Status at Baseline eTable 14. Percentage of Spousal Concordance by the ICVHMs eFigure 1. Participant Flow Diagram eFigure 2. Study Sites and Participant Distribution eFigure 3. Association of Comparison of Numbers of ICVHMs Between Couples and Spousal Diabetes Diagnosis With Incident Diabetes in Men eFigure 4. Association of Comparison of Numbers of ICVH [file jamanetwopen-e2319038-s001.pdf]

Supplemental Online Content

Zhao Z, Cao Q, Lu J, et al. Association of spousal diabetes status and ideal cardiovascular health metrics with risk of incident diabetes among Chinese adults. *JAMA Netw Open.* 2023;6(6):e2319038. doi:10.1001/jamanetworkopen.2023.19038

eAppendix. Supplementary Methods

eTable 1. Data Types and Definitions of Covariates Involved in the Study

eTable 2. Definitions of ICVHM

eTable 3. Association Between Husband's Diabetes Diagnosis and Incident Diabetes in Women

eTable 4. Association Between Wife's Diabetes Diagnosis and Incident Diabetes in Men

eTable 5. Association Between Spousal Diabetes Diagnosis and Incident Diabetes in Models Further Adjusted for Alcohol Consumption

eTable 6. Association Between Spousal Diabetes Diagnosis and Incident Diabetes in Models Replacing Prediabetes Status with Baseline HbA1c Level

eTable 7. Association Between Spousal Diabetes Diagnosis and Incident Diabetes in Multiple Imputation Data Set Imputed for Missing Baseline Information

eTable 8. Association of Individual ICVHMs and Spousal Diabetes Diagnosis With Incident Diabetes in Multiple Imputation Data Set Imputed for Missing Baseline Information

eTable 9. Association of Spousal ICVHMs and Spousal Diabetes Diagnosis With Incident Diabetes in Multiple Imputation Data Set Imputed for Missing Baseline Information

eTable 10. Association of Comparison of Numbers of ICVHMs Between Couples and Spousal Diabetes Diagnosis With Incident Diabetes in Multiple Imputation Data Set Imputed for Missing Baseline Information

eTable 11. Association Between Spousal Diabetes Diagnosis With Incident Diabetes in Multiple Imputation Data Set Imputed for Outcome

eTable 12. Association Between Spousal Diabetes Diagnosis With Incident Diabetes Estimated by Random-Effects Models

eTable 13. Baseline Characteristics of Individuals Categorized by Diabetes Diagnosis at Follow-up and Spousal Diabetes Status at Baseline

eTable 14. Percentage of Spousal Concordance by the ICVHMs

eFigure 1. Participant Flow Diagram

eFigure 2. Study Sites and Participant Distribution

eFigure 3. Association of Comparison of Numbers of ICVHMs Between Couples and Spousal Diabetes Diagnosis With Incident Diabetes in Men

eFigure 4. Association of Comparison of Numbers of ICVHMs Between Couples and Spousal Diabetes Diagnosis With Incident Diabetes in Women

eFigure 5. Association of Individual CVH Score Categories and Spousal Diabetes Diagnosis With Incident Diabetes

eFigure 6. Association of Spousal CVH Score Categories and Spousal Diabetes Diagnosis With Incident Diabetes

eFigure 7. Association of Comparison of CVH Scores Between Couples and Spousal Diabetes

© 2023 Zhao Z et al. *JAMA Network Open.*

Diagnosis With Incident Diabetes

This supplemental material has been provided by the authors to give readers additional information about their work.

eAppendix. Supplementary Methods

Baseline Examination

Clinic visits took place at the community health clinics in the participants' residential area. All clinical visits were scheduled in the morning. Participants were required to fast for at least 10 hours prior to their clinic visits. In addition, they were provided instructions and a container for collecting the first morning spot urine sample before their clinic appointment. Data on sociodemographic information (e.g. education), lifestyle factors (e.g. cigarette smoking, alcohol drinking, sleep habits, and dietary factors), and medical history (e.g., diabetes, cardiovascular disease, and cancer) were obtained by trained study personnel using a standard questionnaire. Physical activity was assessed using the International Physical Activity Questionnaire. Moderate and vigorous physical activity was defined as ≥150 minutes/week of moderate-intensity physical activity, or 75 minutes/week of vigorous-intensity aerobic physical activity, or an equivalent combination of moderate- and vigorous-intensity aerobic activities. We used a food frequency questionnaire to evaluate dietary habit in the previous year. The healthy diet score included the following 7 components: fruits and vegetables ≥4.5 cups/day, fish ≥two 3.5-oz servings/week, livestock and poultry meat: 40-75g/day, sweets/sugar-sweetened beverages ≤450 kcal/week, soy protein ≥25 g/day, milk or yogurt ≥3 cups/week, alcohol ≤ 2 servings per day for men or ≤ 1 serving per day for women.

Body weight, height, waist circumference, and blood pressure were measured according to a standard protocol by trained study nurses. Three blood pressure measurements were obtained with participants in a seated position after five minutes of quiet rest. In addition, participants were required to avoid alcohol, cigarettes, coffee/tea, and exercise for ≥30 minutes before their measurement. An automated electronic device (OMRON Model HEM-752 FUZZY, Omron Company, Dalian, China) was used on the non-dominant arm of participants. One of four cuff sizes (pediatric, regular adult, large, or thigh) was chosen based on each participant's arm circumference. Three blood pressure readings were averaged for analysis. Body-mass index was calculated as body weight in kilograms divided by body height squared in meters.

All participants underwent an oral glucose tolerance test, and plasma glucose was obtained at zero and two hours after the administration of 75 g of glucose. Plasma glucose concentrations were analyzed locally using a glucose oxidase or hexokinase method within two hours after blood sample collection under a stringent quality control program.

Serum samples were aliquoted into 0.5 mL Eppendorf tubes within 2 hours and shipped by air in dry ice to the study central laboratory at the Shanghai Institute of Endocrine and Metabolic Disease. The Laboratory has regularly participated the proficiency-testing program and passed the College of American Pathologists (CAP)'s Laboratory Accreditation Program. Total cholesterol, low-density lipoprotein cholesterol, high-density lipoprotein cholesterol, and triglycerides (TG) were measured using enzymatic methods with an auto-analyzer (ARCHITECT ci16200 System, Abbott Laboratories, Illinois, USA). Fasting insulin was measured using an auto-analyser (ARCHITECT i2000SR System, Abbott Laboratories).

The Hemoglobin Capillary Collection System (Bio-Rad Laboratories, CA, USA) was used to collect finger capillary blood samples in strict accordance with the manufacturer's instructions. Blood specimens prepared using this procedure were stable for up to 4 weeks

© 2023 Zhao Z et al. *JAMA Network Open.*

at 2°C to 8°C. The capillary blood specimens were shipped and stored at 2°C to 8°C until hemoglobin A1c was measured within 4 weeks after collection, which is within the range of the stability according to the manufacturer's instruction. Hemoglobin A1c was measured by high-performance liquid chromatography using the VARIANT II Hemoglobin Testing System (Bio-Rad Laboratories, CA, USA) at the central laboratory in the Shanghai Institute of Endocrine and Metabolic Diseases, which was certified by the US National Glycohemoglobin Standardization Program. The total imprecision, in terms of coefficients of variation (CVs) were 1.66% and 1.85% at HbA1c levels of 5.7% and 9.6% respectively in ourstudy.

Follow-up Visit and Outcome Assessment

During 2014-2016, 193,846 study participants from 20 communities were invited to participate in an in-person follow-up visit. Lifestyle risk factors and medical history were queried by trained staff using a standard questionnaire. Anthropometric and blood pressure measurements, oral glucose tolerance tests, and blood samples were obtained using the same protocol that was used in the baseline examination. If patients were hospitalized or visited an emergency department, their medical records, including medical history, findings on the physical examination, laboratory tests, treatments, and discharge diagnosis, were abstracted by trained staff using a standard form. In addition, photocopies of selected sections of the participant's inpatient record, discharge summary, electrocardiogram, and pathology reports were obtained.

Information on vital status and clinical outcomes were collected from local registries of the National Disease Surveillance Point System and National Health Insurance System. Two members of the outcome adjudication committee independently verified each clinical event, and discrepancies were adjudicated by discussion involving other members of the committee. All members of the committee were unaware of the baseline risk factors of study participants.

Incident diabetes was defined as fasting plasma glucose ≥126 mg/dL, and/or 2-hour post-load plasma glucose ≥200 mg/dL, and/or hemoglobin A1c ≥6.5%, and/or self-reported use of antidiabetic medication during follow-up among participants without diabetes at baseline.[4]

Quality Control

Data collection followed a stringent quality control process. All study staff underwent a 1-week centralized training program on the study protocol and standard operating procedures. Only those who passed a certification assessment were allowed to collect study data. Regular site visits were conducted by the members of the Study Steering Committee and staff members from the Study and Data Coordinating Center at Ruijin Hospital, Shanghai Jiao-Tong University.

All regional laboratories in this study have regularly participated in the proficiency-testing program and achieved the certification of external quality assessment (EQA) for glucose measurement by National Center for Clinical Laboratories of the People's Republic of China. Fasting and 2-hour postload samples were collected using vacuum blood collection tubes containing anticoagulant sodium fluoride and were centrifuged on site within 2 hours of collection. All local study laboratories underwent a 5-day performance standardization process for glucose measurement. Test results were evaluated by experienced laboratory experts independently at the central laboratory and only the local laboratories which passed the standardization program were qualified to perform study plasma glucose tests. During

© 2023 Zhao Z et al. *JAMA Network Open.*

the study, inter-laboratory and intra-laboratory quality control assessments were conducted on each of the testing days. If the laboratory failed the internal quality control assessment, causes were identified, appropriate modifications were applied, and all blood samples were re-tested. Agents for quality control of glucose measurement were provided by Bio-Rad Laboratories, USA. The coefficients of variation for low and high value were < 3.0%.

Lipid profiles were measured at the study central laboratory, which is certified by the College of American Pathologists, strictly following the laboratory's quality control procedures.

All study data were double-entered using a secured, web-based data system. The data managers at the Study and Data Coordinating Center merged the two independent datasets and checked for missing data and unrealistic values and performed crosschecks for inconsistencies. Data queries generated from quality control procedures were sent to participating centers and a timely reply was required. Any deficiencies were addressed through direct feedback with study personnel, and, at times, supplemental trainings on procedures were given if problematic data were observed.

Statistical analysis

The modifying effect of individual ICVHM on the association of spousal diabetes status and individual diabetes was evaluated in stratified analyses by strata of the 8 individual components of ICVHM and the total number of ICVHM components. We also compared the risk estimates in the strata of other risk factors, including age, sex, and family history of diabetes.

The effect of spousal ICVHMs on the association of spousal diabetes status and individual diabetes was estimated in stratified analyses by the total number of ICVHM in spouses.

The effect of discordance of ICVHMs between couples on the association of spousal diabetes status and individual diabetes was investigated in stratified analyses. To compare the CVH status of the couples, we generated 3 groups "Number of individual ICVHM > Number of spousal ICVHM", "Number of individual ICVHM = Number of spousal ICVHM" and "Number of individual ICVHM < Number of spousal ICVHM". These 3 levels reflected the CVH status in a person was better than, equal to, or poorer than his/her spouse, respectively. And the association was tested in two models. Model 1 was unadjusted. Model 2 was adjusted for high school education or above, family history of diabetes, local GDP and urban or not.

To address the validity of the major analysis, a multiple imputation chain-equation was used to impute baseline missing data. Data were assumed to be missing at random. Missing continuous variables were imputed using regression method, and binary variables were imputed by logistic regression model. We generated five imputed datasets with 100th iteration, and analyzed each dataset separately, then combined their results by use of Rubin's method. Besides, to adjust for censoring (loss to follow-up), we further performed a sensitivity analysis using multiple imputation to impute the time-to-event and outcome information of those who lost to follow-up, and tested the associations in the imputed dataset.

© 2023 Zhao Z et al. *JAMA Network Open.*

eTable 1. Data Types and Definitions of Covariates Involved in the Study

Covariate	Data type during analysis	Categorical variable definition or continuous variable unit
Education	Categorical variable	Less education: less than high school (<9 years)<br>Higher education: high school or further (≥9 years)
Family history of diabetes	Categorical variable	Yes: At least one of the first-degree relatives was diagnosed as diabetes<br>No: No one among first-degree relatives was diagnosed as diabetes
Location	Categorical variable	Urban or not
Sex	Categorical variable	/
Local personal income	Continuous variable	Annual household income per capita per resident based on the survey conducted by National Bureau of Statics
Diet score	Continuous variable	/
Systolic blood pressure	Continuous variable	mm Hg
Diastolic blood pressure	Continuous variable	mm Hg
BMI	Continuous variable	kg/m^2
Waist circumstance	Continuous variable	cm
FPG	Continuous variable	mmol/L
2h PG	Continuous variable	mmol/L
HbA1c	Continuous variable	%
Total cholesterol	Continuous variable	mg/dl
LDL-cholesterol	Continuous variable	mg/dl
HDL-cholesterol	Continuous variable	mg/dl
Triglycerides	Continuous variable	mg/dl

BMI, body mass index; HDL, high-density lipoprotein; LDL, low-density lipoprotein; FPG, fasting blood glucose; 2h PG, 2h post-load blood glucose; HbA1c, glycated hemoglobin; ICVHM, ideal cardiovascular health metric.

© 2023 Zhao Z et al. *JAMA Network Open.*

eTable 2. Definitions of ICVHM

CVH metric	Method of measurement	Quantification of CVH metric
Smoking	Self-reported use of cigarettes	Points Tobacco use and secondhand smoke exposure<br>100: Never smoker<br>75: Former smoker, quit ≥5 years<br>50: Former smoker, quit 1–<5 years<br>25: Former smoker, quit <1 year<br>0: Current smoker<br>Subtract 20 points (unless score is 0) for living with active indoor smoker in home
Physical activity	Self-reported physical activity	Points Minutes of moderate or vigorous physical activity per week<br>100: ≥150<br>90: 120–149<br>80: 90–119<br>60: 60–89<br>40: 30–59<br>20: 1–29<br>0: 0
Sleep health	Self-reported average hours of sleep per night	Points Hours of sleep per night<br>100: 7-<9<br>90: 9-<10<br>70: 6-<7<br>40: 5-<6 or ≥10<br>20: 4–<5<br>0: <4
Diet	Self-reported daily intake of items on the MEPA screener, modified according to the dietary habits of Chinese population.	Modified MEPA screener score:<br>(1) 4 points were given for ≥4 servings of vegetables and fruits per day;<br>(2) 1 point was given for ≤3 servings of red meat per week;<br>(3) 1 point was given for ≥1 servings of fish per week;<br>(4) 1 point was given for ≤5 servings of chicken per week;<br>(5) 1 point was given for ≥3 servings of beans per week;<br>(6) 1 point was given for ≤4 servings of cookies or cakes per week;<br>(7) 1 point was given for ≤1 meal at a restaurant per week;<br>(8) 1 point was given for >0 or ≤2 servings of alcohol per day for men and >0 or ≤1

© 2023 Zhao Z et al. *JAMA Network Open.*

		serving of alcohol per day for women;<br>If any condition was not met, a score of '0' was recorded for that item. The total modified MEPA score could range from 0 to 11.<br>Scoring:<br>Points Modified MEPA score (points)<br>100 10-11<br>80 8-9<br>50 6-7<br>25 4-5<br>0 0-3
BMI	Body weight (kilograms) divided by height squared (meters squared)	Metric: BMI (kg/m2)<br>Scoring:<br>Points Level<br>100 <23.0<br>70 23.0-24.9<br>50 25.0-29.9<br>25 30.0-34.9<br>0 ≥35.0
Blood pressure	Objective measurement of SBP and DBP	Points SBP and DBP (mmHg)<br>100: <120/80 (optimal)<br>75: 120-129/<80 (elevated)<br>50: 130-139 or 80-89 (stage 1 hypertension)<br>25: 140-159 or 90-99<br>0: ≥160 or ≥100<br>Subtract 20 points if treated level
Blood lipids	Fasting Plasma non–HDL cholesterol tested at the central laboratory	Points Non-HDL cholesterol (mg/dl)<br>100: <130<br>60: 130-159<br>40: 160-189<br>20: 190-219<br>0: ≥220<br>Subtract 20 points if treated level
Blood glucose	Fasting plasma glucose tested locally, and fasting HbA1c tested at the central laboratory	Points Glycemic status

© 2023 Zhao Z et al. *JAMA Network Open.*

		100: NGR: No history of diabetes and FPG<100 mg/dl (or HbA1c<5.7%)<br>60: Prediabetes: No diabetes and FPG 100-125mg/dl or (or HbA1c 5.7-6.4%)<br>40: Diabetes with HbA1c<7.0%<br>30: Diabetes with HbA1c: 7.0-7.9%<br>20: Diabetes with HbA1c: 8.0-8.9%<br>10: Diabetes with HbA1c: 9.0-9.9%<br>0: Diabetes with HbA1c: ≥10.0%

CVH, cardiovascular health; BMI, body mass index; HDL, high-density lipoprotein; NGR, normal glucose regulation; FPG, fasting plasma glucose; 2h-PG, 2-hour post-load plasma glucose; HbA1c, glycated hemoglobin; SBP, systolic blood pressure; DBP, diastolic blood pressure; ICVHM, ideal cardiovascular health metric; MEPA, Mediterranean Eating Pattern for Americans.

© 2023 Zhao Z et al. *JAMA Network Open.*

eTable 3. Association Between Husband's Diabetes Diagnosis and Incident Diabetes in Women

Husband status	Without diagnosed DM	With diagnosed DM		
		Total	HbA1c<7.0%	HbA1c>=7.0%
Case number	1247 / 16205	187 / 1917	71 / 852	116 / 1065
DM incidence	7.70%	9.75%**	8.33%	10.89%***
HR (95%CI)				
Unadjusted	1	1.22 (1.05-1.42)*	1.08 (0.85-1.37)	1.33 (1.10-1.61)**
Model 1	1	1.20 (1.03-1.40)*	1.00 (0.79-1.28)	1.36 (1.12-1.65)**
Model 2	1	1.15 (0.99-1.34)	1.00 (0.79-1.27)	1.27 (1.05-1.54)*
Model 3	1	1.14 (0.97-1.33)	0.98 (0.77-1.25)	1.26 (1.04-1.53)*
Model 4	1	1.13 (0.97-1.32)	0.98 (0.77-1.24)	1.25 (1.03-1.52)*

DM, diabetes; HR, hazard ratio; HbA1c, glycated hemoglobin. The adjusted variables in the models are consistent with Figure 1.

© 2023 Zhao Z et al. *JAMA Network Open.*

eTable 4. Association Between Wife's Diabetes Diagnosis and Incident Diabetes in Men

Wife status	Without diagnosed DM	With diagnosed DM		
		Total	HbA1c<7.0%	HbA1c>=7.0%
Case number	1317 / 15411	145 / 1288	69 / 602	76 / 686
DM incidence	8.55%	11.26%***	11.46%*	11.08%*
HR (95%CI)				
Unadjusted	1	1.30 (1.10-1.54)**	1.33 (1.04-1.69) *	1.28 (1.01-1.61)*
Model 1	1	1.22 (1.03-1.45)*	1.24 (0.97-1.58)	1.21 (0.96-1.52)
Model 2	1	1.21 (1.01-1.43)*	1.26 (0.98-1.60)	1.16 (0.92-1.47)
Model 3	1	1.19 (1.00-1.41)	1.24 (0.97-1.58)	1.15 (0.91-1.45)
Model 4	1	1.19 (1.00-1.41)	1.25 (0.98-1.59)	1.14 (0.90-1.44)

DM, diabetes; HR, hazard ratio; HbA1c, glycated hemoglobin. The adjusted variables in the models are consistent with Figure 1.

© 2023 Zhao Z et al. *JAMA Network Open.*

eTable 5. Association Between Spousal Diabetes Diagnosis and Incident Diabetes in Models Further Adjusted for Alcohol Consumption

Spouse status	Without diagnosed DM	With diagnosed DM		
		Total	HbA1c<7.0%	HbA1c>=7.0%
Case number	2564 / 31616	332 / 3205	140 / 1454	192 / 1751
DM incidence	8.11%	10.36%***	9.63%*	10.97%***
HR (95%CI)				
Unadjusted	1	1.25 (1.11-1.40)***	1.18 (1.00-1.40)	1.30 (1.12-1.50)***
Model 1	1	1.20 (1.07-1.35)**	1.10 (0.93-1.31)	1.28 (1.11-1.49)**
Model 2	1	1.17 (1.05-1.32)**	1.11 (0.94-1.32)	1.22 (1.06-1.42)**
Model 3+alcohol consumption	1	1.16 (1.03-1.30)*	1.10 (0.92-1.30)	1.21 (1.05-1.41)*
Model 4+alcohol consumption	1	1.15 (1.03-1.30)*	1.10 (0.92-1.30)	1.20 (1.04-1.39)*

DM, diabetes; HR, hazard ratio; HbA1c, glycated hemoglobin. The other adjusted variables in the models are consistent with Figure 1.

© 2023 Zhao Z et al. *JAMA Network Open.*

eTable 6. Association Between Spousal Diabetes Diagnosis and Incident Diabetes in Models Replacing Prediabetes Status with Baseline HbA_{1c} Level

Spouse status	Without diagnosed DM	With diagnosed DM		
		Total	HbA1c<7.0%	HbA1c>=7.0%
Case number	2564 / 31616	332 / 3205	140 / 1454	192 / 1751
DM incidence	8.11%	10.36%***	9.63%*	10.97%***
HR (95%CI)				
Unadjusted	1	1.25 (1.11-1.40)***	1.18 (1.00-1.40)	1.30 (1.12-1.50)***
Model 1	1	1.20 (1.07-1.35)**	1.11 (0.94-1.32)	1.20 (1.04-1.39)*
Model 2	1	1.16 (1.04-1.31)*	1.15 (0.97-1.36)	1.21 (1.04-1.41)*
Model 3	1	1.15 (1.03-1.29)*	1.10 (0.93-1.30)	1.19 (1.03-1.38)*
Model 4	1	1.14 (1.02-1.28)*	1.10 (0.93-1.30)	1.18 (1.02-1.37)*

DM, diabetes; HR, hazard ratio; HbA1c, glycated hemoglobin. The other adjusted variables in the models are consistent with Figure 1.

© 2023 Zhao Z et al. *JAMA Network Open.*

eTable 7. Association Between Spousal Diabetes Diagnosis and Incident Diabetes in Multiple Imputation Data Set Imputed for Missing Baseline Information

Spouse status	Without diagnosed DM	With diagnosed DM		
		Total	HbA1c<7.0%	HbA1c>=7.0%
Case number	2647 / 32675	339 / 3285	141 / 1483	198 / 1802
DM incidence	8.10%	10.32%***	9.50%	10.99%***
HR (95%CI)				
Unadjusted	1	1.24 (1.11-1.39)***	1.16 (0.98-1.37)	1.30 (1.13-1.50)***
Model 1	1	1.19 (1.06-1.33)**	1.08 (0.91-1.28)	1.28 (1.11-1.48)***
Model 2	1	1.16 (1.04-1.30)**	1.09 (0.92-1.29)	1.22 (1.05-1.41)**
Model 3	1	1.15 (1.03-1.29)*	1.08 (0.91-1.28)	1.21 (1.04-1.40)*
Model 4	1	1.14 (1.02-1.28)*	1.08 (0.91-1.28)	1.20 (1.03-1.39)*

DM, diabetes; HR, hazard ratio; HbA1c, glycated hemoglobin. The adjusted variables in the models are consistent with Figure 1.

© 2023 Zhao Z et al. *JAMA Network Open.*

eTable 8. Association of Individual ICVHMs and Spousal Diabetes Diagnosis With Incident Diabetes in Multiple Imputation Data Set Imputed for Missing Baseline Information

Individual status	Incident DM/Total cases (%)		Hazard Ratios (95%CI) for Spouse's status
No. of ICVHMs			Spouse's status: Diagnosed DM with uncontrolled glucose
≤2	1336 / 11754	11.37%	1.35 (1.09,1.68)
3	762 / 8912	8.55%	1.43 (1.10,1.87)
4	553 / 7669	7.21%	1.03 (0.70,1.51)
≥5	335 / 7625	4.39%	1.00 (0.63,1.59)
P for interaction	0.36		

DM, diabetes; HR, hazard ratio; HbA1c, glycated hemoglobin; ICVHM, ideal cardiovascular health metrics. Model was adjusted for age, sex, high school education or above, family history of diabetes, local personal income and urban or not.

© 2023 Zhao Z et al. *JAMA Network Open.*

eTable 9. Association of Spousal ICVHMs and Spousal Diabetes Diagnosis With Incident Diabetes in Multiple Imputation Data Set Imputed for Missing Baseline Information

Spouse's status	Incident DM/Total cases (%)		Hazard Ratios (95%CI) for Spouse's status
No. of ICVHMs			Spouse's status: Diagnosed DM with uncontrolled glucose
≤2	1213 / 14045	8.64%	1.25 (1.03,1.51)
3	731 / 8880	8.23%	1.44 (1.07,1.92)
4	557 / 6774	8.22%	1.28 (0.83,1.99)
≥5	465 / 6241	7.45%	1.30 (0.65,2.63)
P for interaction	0.60		

DM, diabetes; HR, hazard ratio; HbA1c, glycated hemoglobin; ICVHM, ideal cardiovascular health metrics. Model was adjusted for age, sex, high school education or above, family history of diabetes, local personal income and urban or not.

© 2023 Zhao Z et al. *JAMA Network Open.*

eTable 10. Association of Comparison of Numbers of ICVHMs Between Couples and Spousal Diabetes Diagnosis With Incident Diabetes in Multiple Imputation Data Set Imputed for Missing Baseline Information

Discordance of CVH status between couples	Incident DM/Total cases (%)	HR (95%CI) for spouse's diabetes status in model 1	HR (95%CI) for spouse's diabetes status in model 2
Individual CVH number > spouse CVH number	1075 / 16156 (6.65%)	1.20 (0.97-1.48)	1.17 (0.94-1.45)
Individual CVH number = spouse CVH number	647 / 7171 (9.02%)	1.57 (1.16-2.11)	1.56 (1.15-2.11)
Individual CVH number < spouse CVH number	1264 / 12633 (10.00%)	1.77 (1.36-2.31)	1.70 (1.31-2.22)
P for interaction		0.016	0.038

DM, diabetes; HR, hazard ratio; CVH, cardiovascular health. Model 1: unadjusted; Model 2: adjusted for high school education or above, family history of diabetes, local personal income and urban or not.

© 2023 Zhao Z et al. *JAMA Network Open.*

eTable 11. Association Between Spousal Diabetes Diagnosis With Incident Diabetes in Multiple Imputation Data Set Imputed for Outcome

Spouse status	Without diagnosed DM	With diagnosed DM		
		Total	HbA1c<7.0%	HbA1c>=7.0%
Case number	3511 / 43475	444 / 4423	183 / 1960	261 / 2463
DM incidence	8.08%	10.04%***	9.34%	10.60%***
HR (95%CI)				
Unadjusted	1	1.24 (1.11-1.38)***	1.17 (0.98-1.41)	1.29 (1.11-1.49)***
Model 1	1	1.19 (1.07-1.34)**	1.09 (0.91-1.31)	1.27 (1.10-1.48)**
Model 2	1	1.16 (1.03-1.30)*	1.09 (0.91-1.32)	1.21 (1.04-1.40)**
Model 3	1	1.14 (1.02-1.28)*	1.07 (0.89-1.30)	1.20 (1.03-1.39)*
Model 4	1	1.14 (1.02-1.27)*	1.07 (0.88-1.29)	1.19 (1.03-1.38)*

DM, diabetes; HR, hazard ratio; HbA1c, glycated hemoglobin. The adjusted variables in the models are consistent with Figure 1.

© 2023 Zhao Z et al. *JAMA Network Open.*

eTable 12. Association Between Spousal Diabetes Diagnosis With Incident Diabetes Estimated by Random-Effects Models

Spouse status	Without diagnosed DM	With diagnosed DM		
		Total	HbA1c<7.0%	HbA1c>=7.0%
Case number	2564 / 31616	332 / 3205	140 / 1454	192 / 1751
DM incidence	8.11%	10.36%***	9.63%*	10.97%***
OR (95%CI)				
Unadjusted	1	1.24 (1.10-1.40)***	1.15 (0.96-1.38)	1.32 (1.12-1.54)***
Model 1	1	1.16 (1.03-1.32)*	1.06 (0.88-1.27)	1.25 (1.07-1.47)**
Model 2	1	1.15 (1.02-1.31)*	1.07 (0.89-1.30)	1.22 (1.03-1.43)*
Model 3	1	1.15 (1.01-1.31)*	1.07 (0.89-1.29)	1.21 (1.03-1.43)*
Model 4	1	1.15 (1.01-1.31)*	1.08 (0.89-1.30)	1.21 (1.03-1.43)*

DM, diabetes; OR, odds ratio; HbA1c, glycated hemoglobin. The adjusted variables in the models are consistent with Figure 1.

© 2023 Zhao Z et al. *JAMA Network Open.*

eTable 13. Baseline Characteristics of Individuals Categorized by Diabetes Diagnosis at Follow-up and Spousal Diabetes Status at Baseline

	Individuals who did not develop diabetes during follow-up and had spouses who did not have diagnosed diabetes at baseline	Individuals who developed diabetes during follow-up but had spouse who did not have diagnosed diabetes at baseline	Individuals who developed diabetes during follow-up and had spouses with diagnosed diabetes at baseline
N	29052	2564	332
Age, mean (SD), y	55.9 (8.27)	57.9 (7.83) *	60.7 (7.55) *#
Sleep duration, mean (SD), hours/day	8.29 (1.50)	8.17 (1.44) *	8.14 (1.37)
BMI, mean (SD), kg/m^2	24.4 (3.45)	25.6 (3.43) *	26.0 (3.65) *
Systolic BP, mean (SD), mm Hg	130.6 (19.3)	136.7 (19.4) *	138.3 (19.2) *
Diastolic BP, mean (SD), mm Hg	78.4 (10.9)	80.4 (11.1) *	80.2 (11.1) *
FPG, mean (SD), mmol/L	5.42 (0.53)	5.71 (0.59) *	5.68 (0.58) *
2h PG, mean (SD), mmol/L	6.68 (1.64)	7.81 (1.88) *	7.93 (1.74) *
HbA1c, mean (SD), %	5.66 (0.38)	5.84 (0.38) *	5.83 (0.38) *

© 2023 Zhao Z et al. *JAMA Network Open.*

Total Cholesterol, mean (SD), mmol/L	4.87 (1.10)	5.00 (1.12) *	5.10 (1.03) *
LDL-c, mean (SD), mmol/L	2.83 (0.86)	2.93 (0.88) *	3.02 (0.84) *
HDL-c, mean (SD), mmol/L	1.32 (0.36)	1.29 (0.35) *	1.27 (0.32) *
Triglycerides, median (IQR), mmol/L	1.26 (0.91)	1.48 (1.09) *	1.49 (0.98) *
Diet scores, mean (SD)	5.67 (2.10)	5.81 (2.12) *	6.02 (2.13) *
Spouse's FPG, median (IQR), mmol/L	5.50 (0.84)	5.50 (0.84)	7.79 (2.61) *#
Spouse's 2h PG, median (IQR), mmol/L	6.90 (2.80)	6.95 (2.85) *	13.10 (6.1) *#
Spouse's HbA1c, mean (SD), %	5.82 (0.71)	5.83 (0.73)	7.45 (1.51) *#

* Vs Individuals who did not develop diabetes during follow-up and had spouses who did not have diagnosed diabetes at baseline P <0.05; # vs Individuals who developed diabetes during follow-up but had spouse who did not have diagnosed diabetes at baseline P<0.05; BP, blood pressure; BMI, body mass index; HDL, high-density lipoprotein; LDL, low-density lipoprotein; FPG, fasting blood glucose; 2h PG, 2h post-load blood glucose; HbA1c, glycated hemoglobin.

© 2023 Zhao Z et al. *JAMA Network Open.*

eTable 14. Percentage of Spousal Concordance by the ICVHMs

	Diet	Physical activity	Sleep habits	Smoking	BMI	Blood pressure	Blood glucose	Blood lipids
Concordant-nonideal	46.17%	77.09%	34.86%	4.41%	47.70%	59.18%	24.19%	38.90%
Concordant-ideal	26.33%	4.70%	28.03%	30.82%	11.83%	8.16%	32.26%	20.59%
Discordant	27.49%	18.22%	37.11%	64.77%	40.46%	32.66%	43.55%	40.51%

BMI, body mass index; ICVHM, ideal cardiovascular health metrics.

© 2023 Zhao Z et al. *JAMA Network Open.*

eFigure 1. Participant Flow Diagram

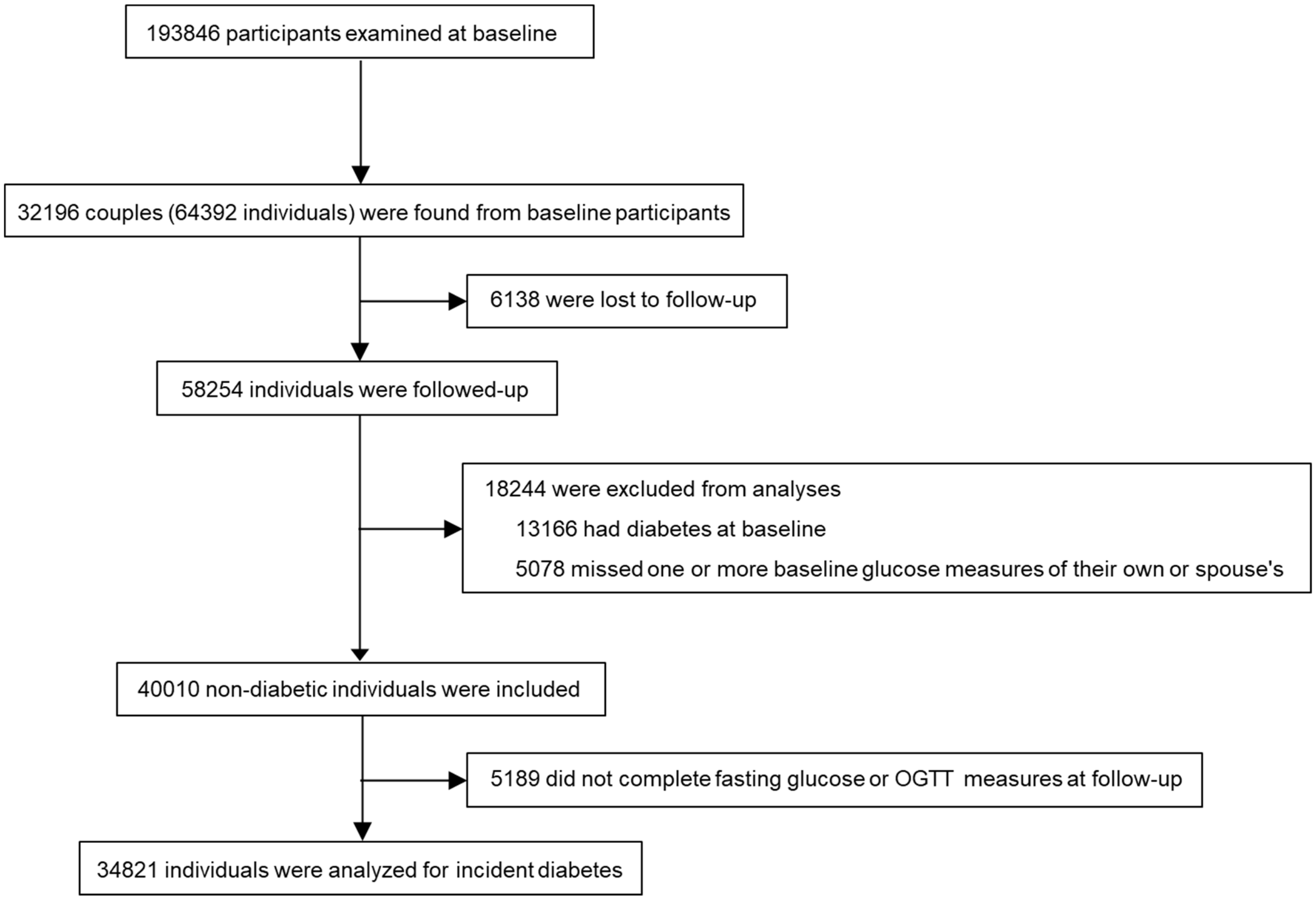

OGTT, Oral glucose tolerance test.

© 2023 Zhao Z et al. *JAMA Network Open.*

eFigure 2. Study Sites and Participant Distribution

A.

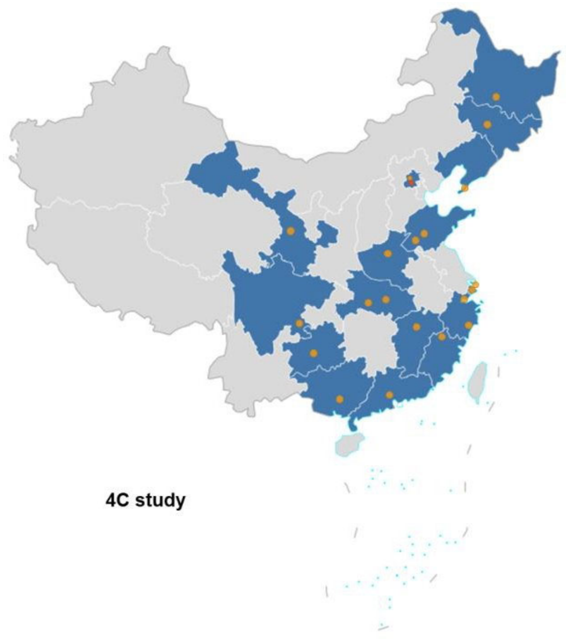

B.

No.	Name of provincial-level region	Name of city/district	Number of participants
1	Zhejiang	Jiashan	3197
2	Sichuan	Luzhou	1610
3	Shanghai	Chongming	2425
4	Guangxi	Nanning	934
5	Guizhou	Guiyang	2052
6	Heilongjiang	Haerbing	812
7	Gansu	Lanzhou	2162
8	Jilin	Changchun	1037
9	Henan	Zhengzhou	2088
10	Guangdong	Guangzhou	1411
11	Jiangxi	Nanchang	2439
12	Shandong	Taian	1311
13	Liaoning	Dalian	1648
14	Zhejiang	Wenzhou	931
15	Fujian	Wuyishan	1712
16	Hubei	Yichang	1757
17	Hubei	Tianmen	152
18	Beijing	Shijingshan	2778
19	Shandong	Jining	1178
20	Shanghai	Jiading	3187

A. The China Cardiometabolic Disease and Cancer Cohort (4C) Study involved 20 study sites from 16 provincial-level regions. B. The participants distribution of the study.

© 2023 Zhao Z et al. *JAMA Network Open.*

eFigure 3. Association of Comparison of Numbers of ICVHMs Between Couples and Spousal Diabetes Diagnosis With Incident Diabetes in Men

		HR (95%CI) for Spouse's Glucose Status: Diagnosed DM with uncontrolled glucose			
Discordance of CVH status between couples	Incident DM/Total cases (%)	Model 1		Model 2	
No. of Individual ICVHM > No. of Wife's ICVHM	306 / 4347 (7.04%)	1.20 (0.79-1.80)		1.12 (0.74-1.69)	
No. of Individual ICVHM = No. of Wife's ICVHM	292 / 3527 (8.28%)	1.20 (0.73-1.96)		1.13 (0.69-1.86)	
No. of Individual ICVHM < No. of Wife's ICVHM	864 / 8825 (9.79%)	1.68 (1.19-2.37)		1.58 (1.12-2.23)	
P for interaction		0.21		0.24	

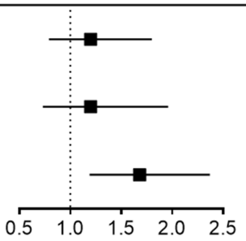

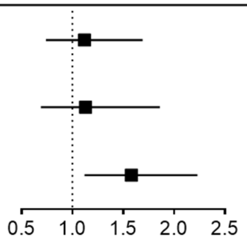

CVH, cardiovascular health; DM, diabetes mellitus; ICVHM, ideal cardiovascular health metrics. Model 1: unadjusted; Model 2: adjusted for high school education or above, family history of diabetes, local personal income and urban or not.

© 2023 Zhao Z et al. *JAMA Network Open.*

eFigure 4. Association of Comparison of Numbers of ICVHMs Between Couples and Spousal Diabetes Diagnosis With Incident Diabetes in Women

Discordance of CVH status between couples	Incident DM/Total cases (%)	HR (95%CI) for Spouse's Glucose Status: Diagnosed DM with uncontrolled glucose — Model 1	HR (95%CI) for Spouse's Glucose Status: Diagnosed DM with uncontrolled glucose — Model 2
No. of Individual ICVHM > No. of Husband's ICVHM	728 / 11283 (6.45%)	1.20 (0.93-1.55)	1.19 (0.92-1.54)
No. of Individual ICVHM = No. of Husband's ICVHM	336 / 3408 (9.86%)	1.95 (1.33-2.85)	2.06 (1.40-3.03)
No. of Individual ICVHM < No. of Husband's ICVHM	370 / 3431 (10.78%)	1.86 (1.21-2.86)	1.95 (1.27-3.01)
P for interaction		0.03	0.02

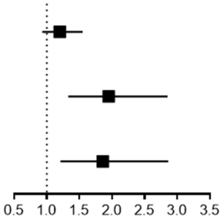

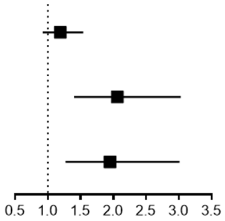

CVH, cardiovascular health; DM, diabetes mellitus; ICVHM, ideal cardiovascular health metrics. Model 1: unadjusted; Model 2: adjusted for high school education or above, family history of diabetes, local personal income and urban or not.

© 2023 Zhao Z et al. *JAMA Network Open.*

eFigure 5. Association of Individual CVH Score Categories and Spousal Diabetes Diagnosis With Incident Diabetes

Individual CVH Score categories	Incident DM/Total cases		Hazard Ratios (95%CI) for Spouse's status<br>Spouse's status: Diagnosed DM with uncontrolled glucose
low	588 / 4245	13.85%	1.42 (1.03,1.95)
moderate	2206 / 28166	7.83%	1.23 (1.04,1.46)
high	102 / 2410	4.23%	1.38 (0.64,2.98)
**P for interaction**	0.87		

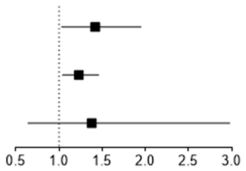

Model was adjusted for age, sex, high school education or above, family history of diabetes, local personal income and urban or not. CVH, cardiovascular health.

© 2023 Zhao Z et al. *JAMA Network Open.*

eFigure 6. Association of Spousal CVH Score Categories and Spousal Diabetes Diagnosis With Incident Diabetes

Spouse's CVH Score categories	Incident DM/Total cases		Hazard Ratios (95%CI) for Spouse's status<br>Spouse's status: Diagnosed DM with uncontrolled glucose
low	1097 / 12739	8.61%	1.24 (1.03,1.49)
moderate	1207 / 14368	8.40%	1.47 (1.11,1.94)
high	592 / 7714	7.67%	1.26 (0.62,2.53)
**P for interaction**	0.48		

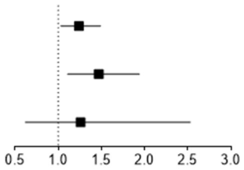

Model was adjusted for age, sex, high school education or above, family history of diabetes, local personal income and urban or not. CVH, cardiovascular health; DM, diabetes mellitus.

© 2023 Zhao Z et al. *JAMA Network Open.*

eFigure 7. Association of Comparison of CVH Scores Between Couples and Spousal Diabetes Diagnosis With Incident Diabetes

			HR (95%CI) for Spouse's Glucose Status: Diagnosed DM with uncontrolled glucose	
**Discordance of CVH between couples**	Incident DM/Total cases (%)		Model 1	Model 2
Individual CVH Score Category < Spouse's CVH Score Category	542 / 4632	(11.70%)	2.00 (1.29-3.09)	1.92 (1.24-2.99)
Individual CVH Score Category = Spouse's CVH Score Category	1832 / 22752	(8.05%)	1.45 (1.18-1.78)	1.44 (1.17-1.77)
Individual CVH Score Category > Spouse's CVH Score Category	522 / 7437	(7.02%)	1.25 (0.98-1.60)	1.18 (0.92-1.52)
P for interaction			0.22	0.28

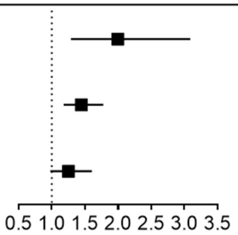

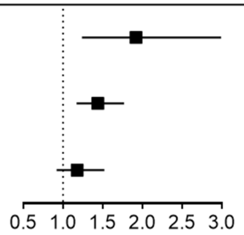

Model 1: unadjusted; Model 2: adjusted for high school education or above, family history of diabetes, local personal income and urban or not. CVH, cardiovascular health; DM, diabetes mellitus.

© 2023 Zhao Z et al. *JAMA Network Open.*
