# Supplementary material for: Association of Spousal Diabetes Status and Ideal Cardiovascular Health Metrics With Risk of Incident Diabetes Among Chinese Adults
Source: JAMA Netw Open. 2023 Jun 23;6(6):e2319038. doi: 10.1001/jamanetworkopen.2023.19038 (PMC10290251; doi:10.1001/jamanetworkopen.2023.19038)
Supplement: Supplement 2. — Data Sharing Statement [file jamanetwopen-e2319038-s002.pdf]

Data Sharing Statement

Zhao. Association of Spousal Diabetes Status and Ideal Cardiovascular Health Metrics With Risk of Incident Diabetes Among Chinese Adults. *JAMA Netw Open*. Published June 23, 2023.
doi:10.1001/jamanetworkopen.2023.19038

Data
Data available: No

Additional Information
Explanation for why data not available: The data and a data dictionary could be open with our access and private protection policy was confirmed and procedures were completed.Please sent application mail to zzybrad@hotmail.com.
